# Study on heat transfer and pressure steady-state characteristics of a floating nozzle under a moving wall

**DOI:** 10.1038/s41598-024-62024-z

**Published:** 2024-05-18

**Authors:** Zhihui Liu, Jiahao Zhang, Zhijian Zhang

**Affiliations:** 1 Present Address: College of Mechanical and Energy Engineering, Shaoyang University, Shaoyang, 422000 China; 2 Hunan Provincial Key Laboratory of Intelligent Manufacturing of High Efficiency Power System, Shaoyang University, Shaoyang, 422000 China

**Keywords:** Suspension nozzle, Moving wall, Flow structure, Heat transfer characteristic, Pressure characteristic, Engineering, Mechanical engineering

## Abstract

This work considers the flow field as two-dimensional turbulent flow and studies the steady-state properties of heat transfer and the pressure of the suspension nozzle. An adiabatic wall parallel to the moving wall and two slit entrances at either end of the adiabatic wall make up the rectangular flow field. The SST $k - \omega$ turbulence model is used in the turbulence computation. Both qualitative and quantitative analyses are conducted on the distribution of the flow field, temperature field, local Nusselt number, local pressure coefficient, average Nusselt number, and average pressure coefficient under various combination conditions. The findings indicate that when the suspension nozzle's flow field varies greatly, wall-jet velocity ratio is 0.1. A rise in Jet inclination angle is not helpful for the wall's suspension, and it has minimal effect on the flow field. The flow field is greatly influenced by separation space-slit width ratio. Larger separation space-slit width ratio values are advantageous for the wall's heat transmission but unfavorable for the wall's suspension. The flow field is most influenced by wall-jet velocity ratio. The wall's ability to convey heat is stronger the higher the wall-jet velocity ratio, but its ability to support weight falls.

## Introduction

**Figure 1:**
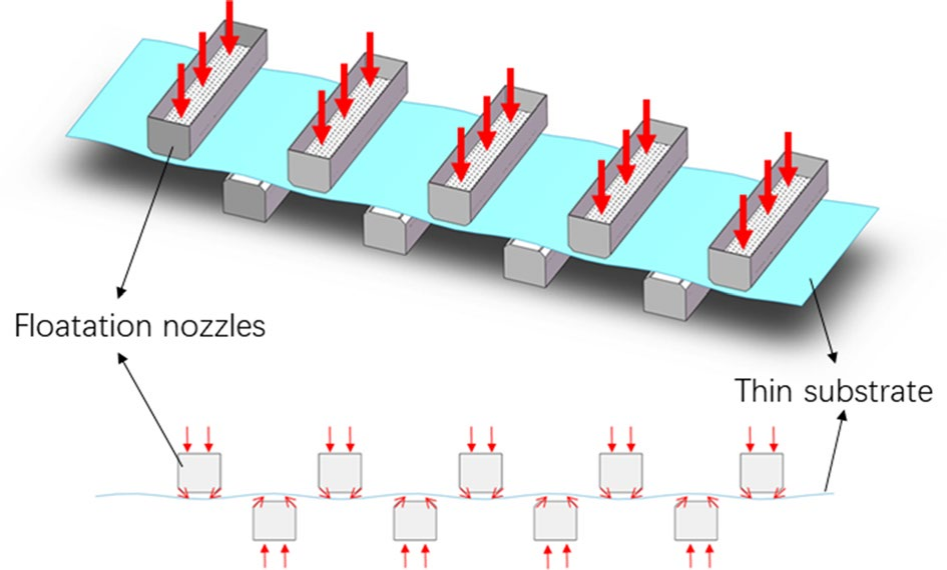
Principle of operation for the suspension nozzle system.

Currently, there are phenomena like rolling on both sides of the substrate and uneven drying of the substrate when the suspension nozzle is used to dry the substrate. These phenomena have a significant impact on the substrate's drying quality and efficiency. Stable suspension and quick drying are two key factors that affect the substrate's drying quality and efficiency. These two factors really involve the substrate's wall pressure distribution and wall heat transmission. The suspension nozzle is essentially a two-slot jet system since the ratio of slot spacing (b) to slot width ($w$) is significantly larger than the ratio of separation distance (h) to slot width ($w$)^1^. When using suspension nozzles as opposed to conventional slit or circular hole nozzles, there is an "air cushion" in between the two slits that helps suspend the thin substrate and prevent substrate scratches and dropping issues during the drying process. The red arrow in Fig. 1 indicates the direction of air flow and illustrates the operation of the floating nozzle system. Numerous real-world uses for this procedure exist, including the production of tempered glass, lithium battery electrode drying, material processing, electronic cooling, and nuclear engineering equipment.

The impingement jet is a crucial fundamental property of the suspension nozzle's pressure and heat transfer properties. Despite a large amount of study on the impingement jet, complicated flow phenomena are present in its several typical locations. For instance, the flow line's curvature varies significantly. Research on flow separation and reattachment as well as the creation, growth, crushing, and merging of vortex formations is still ongoing. In 1989, Polat et al.^2^ provided an extensive overview of the numerical analysis of heat transfer and impinging jet flow. Subsequently, some researchers conducted numerical simulation studies on the impact of the slit on the solid wall and studied the heat transfer characteristics of the impact jet from different angles, and the results were in good agreement with the experimental data^3–5^. In recent decades, a large number of numerical and experimental studies have also been conducted on the impact of inclined circular jets^6–9^. They believe that the location of the stationary point and the maximum heat transfer depend on the tilt angle of the jet, and the high Reynolds number (${\text{Re}}$) also plays an important role. On the other hand, there has been less research done on the heat transmission of oblique jet impact on the surface than vertical jet impact. Double-inclined jets may have a more significant impact on the heat transfer surface in specific technical applications. As a result, some studies find that the effect of double-inclined jets on solid walls is a crucial subject. Research^10^ demonstrates that impacting the hot wall with many slots can enhance the target wall's heat transfer more than doing so with only one hole. By using CFD, Noakes et al.^11^ determined the heat transfer capacities of double- and triple-slit suspension nozzles. The findings indicate that the triple-slit suspension nozzles have a higher heat transfer capacity than the double-slit suspension nozzles. Literature^1^ studies the effects of dip angles in different directions on the flow and heat transfer characteristics of double-groove jets at different Reynolds numbers. The results show that, with the increase in Reynolds number, when $\alpha$ = π/4, the divergent inclined jet has better heat transfer, and when $\alpha$ > π/4 or $\alpha$ < π/4, the vertical jet has the best heat transfer. The heat transfer of divergent inclined jets is greater than that of convergent inclined jets. However, when the ratio of slot spacing to slot width is smaller than that of separation distance to slot width studied in literature^1^, it is still unknown whether the heat transfer situation of the suspension nozzle is the same. In addition, only a few papers related to suspension nozzles focus on pressure distribution on fixed walls^12^ and numerical methods^13–15^.

The influence of wall movement on flow structure cannot be ignored. In the past decade, a number of numerical simulations and experimental studies have provided a better understanding of the effect of wall motion on the flow structure of slit nozzles. Senter et al.^16^ experimentally measured the flow field of the slit nozzle under the combination of different ${\text{Re}}$ and $Rsj$ (the ratio of wall velocity $u_{s}$ to jet velocity $u_{j}$) when $h/w$ remained unchanged. The experimental results showed that when $Rsj$ < 0.25, the flow field of the slit nozzle basically did not change. Therefore, $Rsj$ = 0.25 is the critical value for significant changes in the flow field. The same critical value was obtained by Sharif et al.^17^ and Benmouhoub et al.^4^. However, the research results of Chattopadhyay et al.^18^ and Chattopadhyay et al.^19^ show that the critical value $Rsj$ is 0.1 for significant changes in flow field. Benmouhoub et al.^20^ analyzed the influence of $Rsj$ on the flow field of the inclined slit nozzle and pointed out that by changing the inclination angle of the slit nozzle, the position of the stagnation point could be controlled to control the flow field mode of the nozzle. The above studies are based on the research results of the slit nozzle, and whether the $Rsj$-critical value of the suspension nozzle is the same is unknown. In addition, Kadiyala et al.^21^ used a numerical simulation method to predict the ${\text{Re}}$ range of the laminar flow region, transition flow region, and turbulent flow region of the slit nozzle when $h/w$ = 2 and $Rsj$ varied between 0 and 6, and the results showed that the ${\text{Re}}$ range of the slit nozzle layer to turbulent transition was 400–3000.

The wall motion also has a great influence on the heat transfer and pressure characteristics. The research results of Huang et al.^22^ show that the distribution of $Nu$ (Nusselt number) in suspension nozzle area 2 increases with the increase of $Rsj$, and when $Rsj$ < 0.05, the local distribution of $Nu$ on the wall basically does not change, while the distribution of $Cp$ (Pressure coefficient) is basically not affected by $Rsj$. However, Sharif et al.^17^ studied the influence of wall movement on heat transfer and found that when $Rsj$ < 0.1, the local $Nu$ distribution of the wall basically did not change. In addition, some scholars also studied the influence of the of the law of wall movement on average $Nu$ ($\overline{Nu}$) and average $Cp$ ($\overline{Cp}$). Chattopadhyay et al.^23^ used LES (large eddy simulation) to analyze the influence law of different $Rsj$ on the distribution of wall $\overline{Nu}$ when $h/w$ and ${\text{Re}}$ remain unchanged, and the results showed that when $Rsj$ < 1.2, $\overline{Nu}$ increases with $Rsj$; when $Rsj$ > 1.2, $\overline{Nu}$ decreases with the increase of $Rsj$. Adiyala et al.^24^, aiming to maximize $\overline{Nu}$ and using parameters such as $h/w$ and $Rsj$ as design variables, adopted a neural network model and a miniature genetic algorithm to obtain optimal solutions under different ${\text{Re}}$. Benmouhoub et al.^20^ proposed the optimal tilt angle of the slit nozzle for different $Rsj$ to achieve the best heat transfer mode. Aldabbag et al.^25^ analyzed $Rsj$'s study on the heat transfer characteristics of an array of square nozzles and found that the distribution of wall $Nu$ would present a periodic oscillation state regardless of whether the wall was at rest or in motion. Li^26^, Li^27^, Ma^28^, et al. analyzed the flow field of the suspension nozzle and obtained better uniformity of the flow field and pressure of the suspension nozzle through structural improvement. However, their study did not consider the role of the wall surface or the heat exchange characteristics with the wall surface, which provided a very limited reference for the actual drying process.

In summary, although a large number of studies have analyzed the influence of wall movement on the flow structure, heat transfer, and pressure of the slit nozzle, the influence of wall movement on the suspended nozzle is still unclear due to the different structures of the suspended nozzle and the slit nozzle, which may make the influence of wall movement on the suspended nozzle more complicated. At present, there are a few reports on the effect of wall motion on the flow structure and heat transfer characteristics of suspension nozzles. The flow structure and heat transfer capacity of suspension nozzles are completely different from those of slit nozzles under different separation spacing to slit width ratios ($h/w$), different jet inclination angles ($\alpha$), and different wall to jet velocity ratios ($Rsj$). Therefore, this study attempts to fill this literature gap. Through numerical simulation, this paper studied the effects of different $\alpha$ and $h/w$ on the flow structure, heat transfer, and pressure steady-state characteristics of the suspension nozzle under the moving wall surface, which is helpful to find out the influence of key parameters of the suspension nozzle on the drying process of the substrate, and the research results can provide a theoretical basis for the design and transformation of the suspension nozzle in this field.

## Numerical methods

### Control equation

Since the width-direction ratio of the suspension nozzle size to the slit is greater than 100, the edge effect can be disregarded, and the suspension nozzle's flow and heat transfer mode can be reduced to a two-dimensional flow that is represented using a two-dimensional Cartesian coordinate system. The flow field and heat transfer will eventually approach a quasi-steady state as they evolve. The following are the mass, momentum, and energy conservation equations for the steady incompressible flow that do not account for viscous dissipation:

1 $$\frac{{\partial u_{i} }}{{\partial x_{i} }} = 0$$

2 $$\rho \frac{{\partial u_{i} u_{j} }}{{\partial x_{j} }} = - \frac{\partial P}{{\partial x_{i} }} + \frac{\partial }{{\partial x_{j} }}\left[ {\mu \left( {\frac{{\partial u_{i} }}{{\partial x_{j} }} + \frac{{\partial u_{j} }}{{\partial x_{i} }}} \right) - \rho \overline{{u_{i} u_{j} }} } \right]$$

3 $$\rho u_{j} \frac{\partial T}{{\partial x_{j} }} = \frac{\partial }{{\partial x_{j} }}\left[ {\frac{\mu }{\Pr }\frac{\partial T}{{\partial x_{j} }} - \rho \overline{{Tu_{j} }} } \right]$$

where $u$, $P$, and $T$ represent flow velocity, pressure, and temperature, respectively; $\Pr$ is the Prandtl number of air; and $\rho \overline{{u_{i} u_{j} }}$ and $\rho \overline{{Tu_{j} }}$ represent turbulent stress and turbulent heat flux. In view of the reliability of the SST $k - \omega$ model in predicting nozzle flow field and heat transfer^29–31^, this study adopts the SST $k - \omega$ turbulence model to model turbulent stress and turbulent heat flux in Eqs. 2 and 3:

4 $$\frac{\partial }{{\partial x_{i} }}(\rho ku_{i} ) = \frac{\partial }{{\partial x_{j} }}\left( {\Gamma_{k} \frac{\partial k}{{\partial x_{j} }}} \right) + G_{k} - Y_{k} + S_{k}$$

5 $$\frac{\partial }{{\partial x_{i} }}(\rho \omega u_{i} ) = \frac{\partial }{{\partial x_{j} }}\left( {\Gamma_{\omega } \frac{\partial k}{{\partial x_{j} }}} \right) + G_{{_{\omega } }} - Y_{{_{\omega } }} + D_{{_{\omega } }} + S_{{_{\omega } }}$$

where $k$ and $\omega$ represent turbulent kinetic energy and specific dissipation rate, respectively. $G_{k}$, $Y_{k}$, and $S_{k}$ represent the turbulent kinetic energy generation term, dissipation term, and source term, respectively. $G_{\omega }$, $Y_{\omega }$, $D_{\omega }$, and $S_{\omega }$ represent the production term, dissipation term, cross-diffusion term, and source term of the specific dissipation rate, respectively. $\Gamma_{k}$ and $\Gamma_{\omega }$ represent the effective diffusion coefficients of turbulent kinetic energy and specific dissipation, respectively.

### Boundary conditions and solution methods

**Figure 2:**
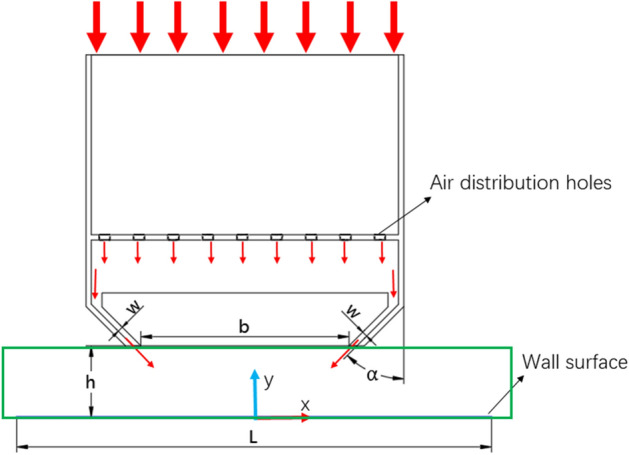
Schematic diagram of suspension nozzle and wall structure.

The uniform flow hole in the suspension nozzle will cause the gas to become uniform once it enters, and the gas flow inside the nozzle has minimal effect on the wall's pressure and heat transfer properties. As a result, the state of the gas following its ejection from the suspension nozzle is the main subject of this investigation. The flow field area to be investigated in this study is illustrated by the inside of the green box line in Fig. 2, which depicts the schematic plan of a suspension nozzle and the wall it controls. Table 1 lists the important parameter.

**Table 1:** Main parameters.

Parameter	Name	Reference value
h	Separation interval	6 mm、12 mm
w	Slit width	1.5 mm
b	Slit spacing	70 mm
$\alpha$	Jet inclination	30°, 45°, 60°
L	The suspension nozzle controls the distance in the x direction	150 mm

**Figure 3:**
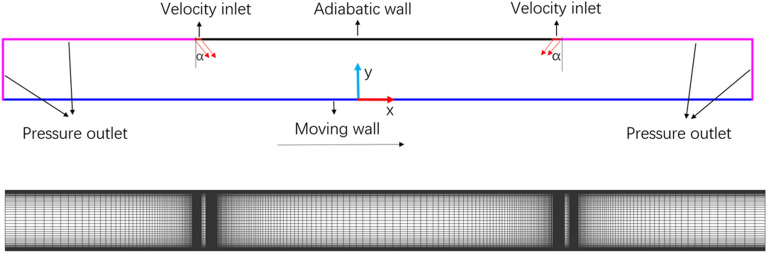
Two-dimensional model and computational grid.

The calculation domain was discretized using a non-uniform quadrilateral mesh; as seen in Fig. 3, the mesh was encrypted close to the adiabatic wall, constant temperature wall, and velocity entry. The left and right air vents' velocity inlet boundary conditions were implemented, and the inlet's turbulence intensity ($TI$) was fixed at 5%^32^. A summary of the boundary conditions can be found in Table 2.

6 $$TI = \sqrt {\frac{2k}{{3u_{j}^{2} }}}$$

where $k$ is turbulent kinetic energy, $u_{j}$ is the jet inlet velocity.

**Table 2:** Summary of boundary conditions.

Boundary condition	Description
Left velocity entry	$u_{x} = u_{j} \sin \alpha$, $u_{y} = { - }u_{j} \cos \alpha$, $TI = 5\%$, $T = T_{h} = 40\; \circ {\text{C}}$
Right velocity entry	$u_{y} = { - }u_{j} \cos \alpha$, $u_{y} = - u_{j} \cos \alpha$, $TI = 5\%$, $T = T_{h} = 40\; \circ {\text{C}}$
Pressure outlet	$\partial u_{x} /\partial x = 0$, $u_{x} = 0$, $P = 0$,$\partial T/\partial x = 0$
Insulated wall	$u_{x} = 0$, $u_{y} = 0$,$\partial T/\partial y = 0$
Moving wall (constant temperature wall)	$u_{x} = u_{j} \times R_{sj}$, $u_{y} = 0$,$T = T_{c} = 25\; \circ {\text{C}}$

The discretized pressure–velocity coupling equation is solved via the SIMPLEC method; the nodal Green-Gauss method processes the gradient term; the central difference method and second-order upwind scheme discretize the diffusion and convection terms; and the discrete continuous equilibrium method in the PRESTO scheme calculates the in-plane "interleaving" pressure of the "interleaving" control body. Because the flow curvature of the suspension nozzle changes widely, the PRESTO format^33^ is advised for flows in naturally convective, spinning, and substantially curved locations. The PRESTO format is utilized to approximate the pressure term. The mass, momentum, turbulence, and energy equations' convergence residuals are all adjusted to 10^–5^, and the flow field simulation studies in this work are finished by FLUENT 2022^33^.

## Model verification

### Grid independence check

In this study, $Rsj$ is between 0 and 1, and $h/w$ is set at 4 and 8, respectively. The grid independence check is chosen for $h/w$ = 8 and $Rsj$ = 1 in order to minimize the impact of the number of grids on the overall simulation process and guarantee the validity of the simulation findings.

Formula 7 illustrates the relationship between the Reynolds number and jet inlet velocity. The Reynolds number directly influences the jet inlet velocity.

7 $${\text{Re}} = \frac{{\rho u_{j} w}}{\mu }$$

where $\rho$ and $\mu$ represent the air density and dynamic viscosity, $u_{j}$ is the jet inlet velocity, and $w$ is the slit width. All calculations are performed under ${\text{Re}}$ = 6000.

**Figure 4:**
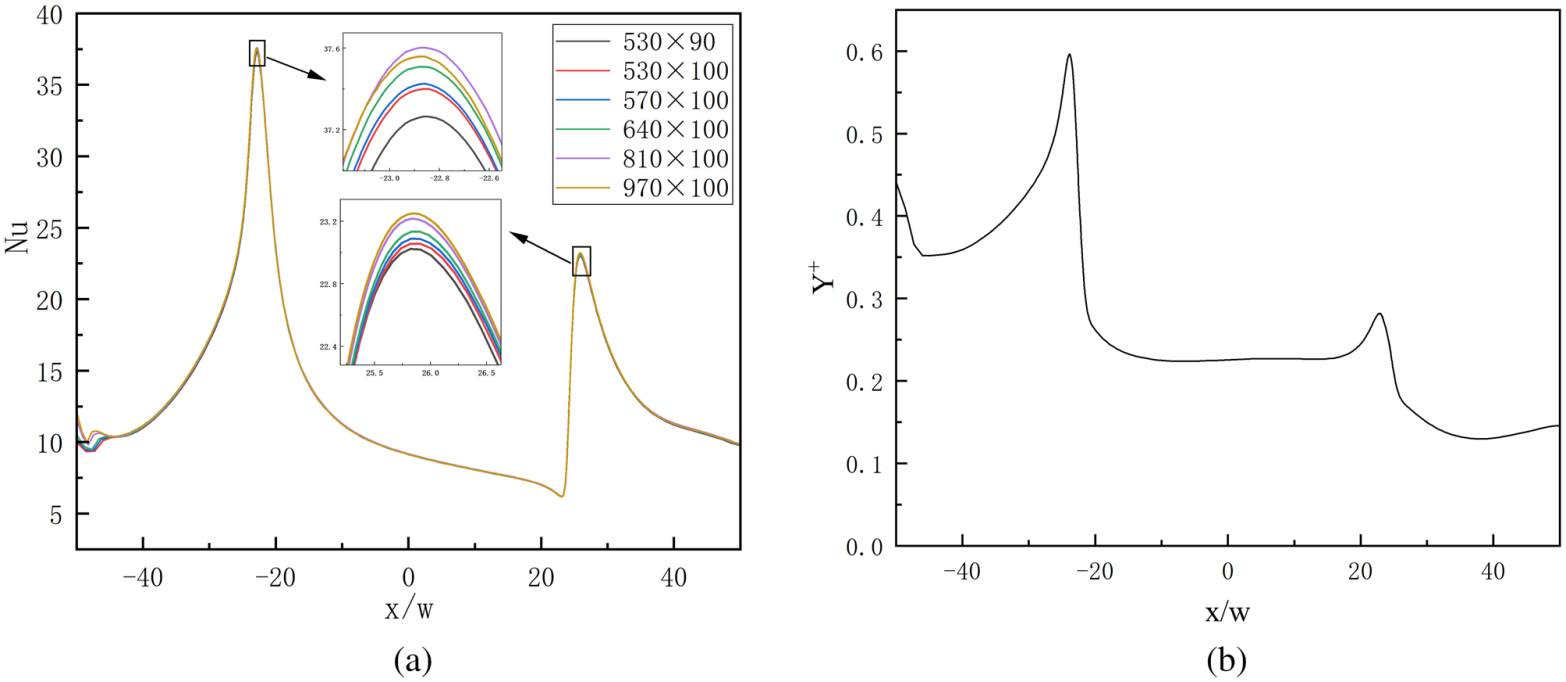
Grid validation and $y^{ + }$ values.

Figure 4a displays the computation results for six mesh sizes under ${\text{Re}}$ = 6000, $Rsj$ = 1, and $h/w$ = 8. The greatest and lowest values of local $Nu$ on the wall essentially remain constant when the grid is 530 × 100, as the figure illustrates. For this reason, the 530 × 100 grid is utilized in the calculation that follows.

The viscous bottom layer of the wall and the buffer layer must be integrated in order for the $\omega$ equation in the SST $k - \omega$ turbulence model to determine the definite solution condition of $y^{ + }$. Because of this, the wall's $y^{ + }$ value must be smaller than 1 in order for at least one node to be placed in the viscous bottom layer of the grid. The wall's $y^{ + }$ curve when using the 530 × 100 grid distribution is depicted in Fig. 4b. This paper's 530 × 100 grid distribution can satisfy the needs of a subsequent simulation because all calculation domains' $y^{ + }$ values are less than 1.

### Model validation analysis

With the exception of a few minor characteristics, such as the placement of the air intake, the experimental boundary conditions employed in this investigation are essentially the same as those found in the literature^22^. We can more readily determine which turbulence model is more accurate by comparing its output with the experimental data, as this allows us to cross-check the model's correctness.

**Figure 5:**
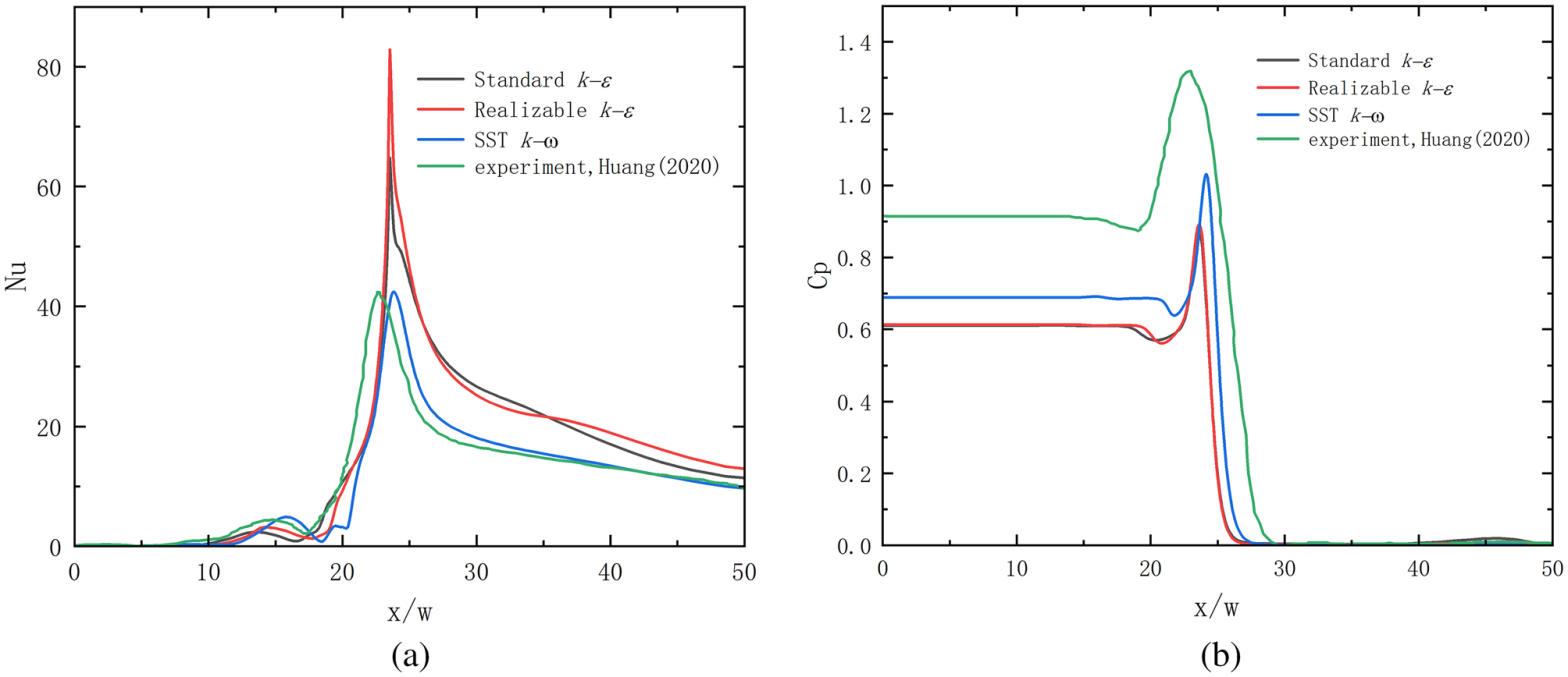
Model comparison verification.

The $Nu$ and $Cp$ findings of the SST $k - \omega$ model are compared to the experimental results of references^22^ in Fig. 5a,b. Additionally, the prediction results of two widely used turbulence models with high prediction accuracy—the Standard $k - \varepsilon$ and Realizable $k - \varepsilon$ models—are displayed. To guarantee that the simulation results' boundary conditions match those of the experiment results, all of the comparative working conditions are $Rsj$ = 0, $h/w$ = 4, $\alpha$ = 45°, and ${\text{Re}}$ = 6000. In this working condition, $Nu$ and $Cp$ are both left and right symmetric images. As a result, the comparison diagram only includes local $Nu$ and $Cp$ in the range 0 < $x/w$ < 50. The Standard $k - \varepsilon$ and Realizable $k - \varepsilon$ models overestimate the experimental findings in the 24 < $x/w$ < 50 region, significantly overestimate the experimental data in the peak location, and underestimate the experimental results in the 10 < $x/w$ < 24 region, as can be observed from Figs. 5a. Nonetheless, the SST $k - \omega$ model exhibits a marginally smaller peak location than the experimental results during the range of 10 < $x/w$ < 24. It then progressively aligns with the experimental results within the range of 24 < $x/w$ < 50. The peak value of the SST $k - \omega$ model is slightly displaced to the right on the $Nu$ curve because the air inlet's location in this model is slightly different from that in the comparative experiment.

Figure 5b shows SST $k - \omega$, Realizable $k - \varepsilon$, and Standard $k - \varepsilon$ models all underestimate the wall's local $Cp$ value. The primary cause is that the jet contains intricate fluid flow phenomena that make it challenging for the CFD model to predict the peak of wall pressure. These phenomena include flow line curvature change, flow separation and reattachment, vortex formation and breakage, and the inherent flaws in the RANS model (many empirical constants, time equality assumptions, etc.). Literature^22^ also noted that the discrepancy between the simulation and experimental results could be attributed to the relatively large ratio between the experimental pressure probe size and slit size; however, the three models' trends matched the experimental results. While the Standard $k - \varepsilon$ and Realizable $k - \varepsilon$ models underestimate the experimental data as well, the SST $k - \omega$ model does so more closely. In conclusion, compared to the Standard $k - \varepsilon$ and Realizable $k - \varepsilon$ models, the SST $k - \omega$ model can more closely match the actual data. For the purpose of the following simulation, the SST $k - \omega$ model is used in this study.

## Results and discussion

### Influence of $h/w$, $\alpha$, and $Rsj$ on flow field and temperature field

**Figure 6:**
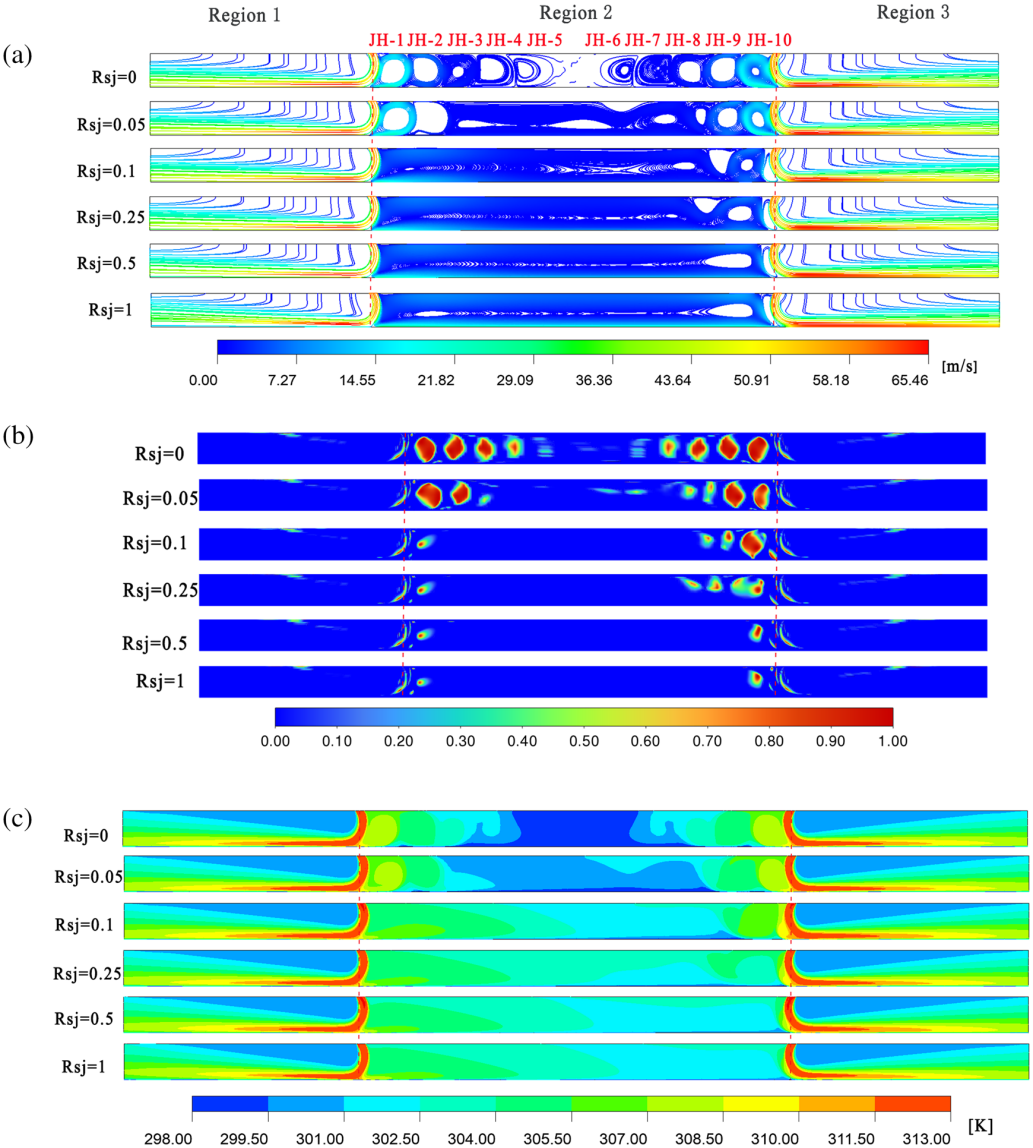
When $\alpha$ = 30°, $h/w$ = 4, velocity flow diagram, $Q$ cloud diagram, and temperature distribution cloud diagram. Ansys Fluent2022, available at https://www.ansys.com/zh-cn/products/fluids/ansys-fluent, simulates and post-processes all pictures.

**Figure 7:**
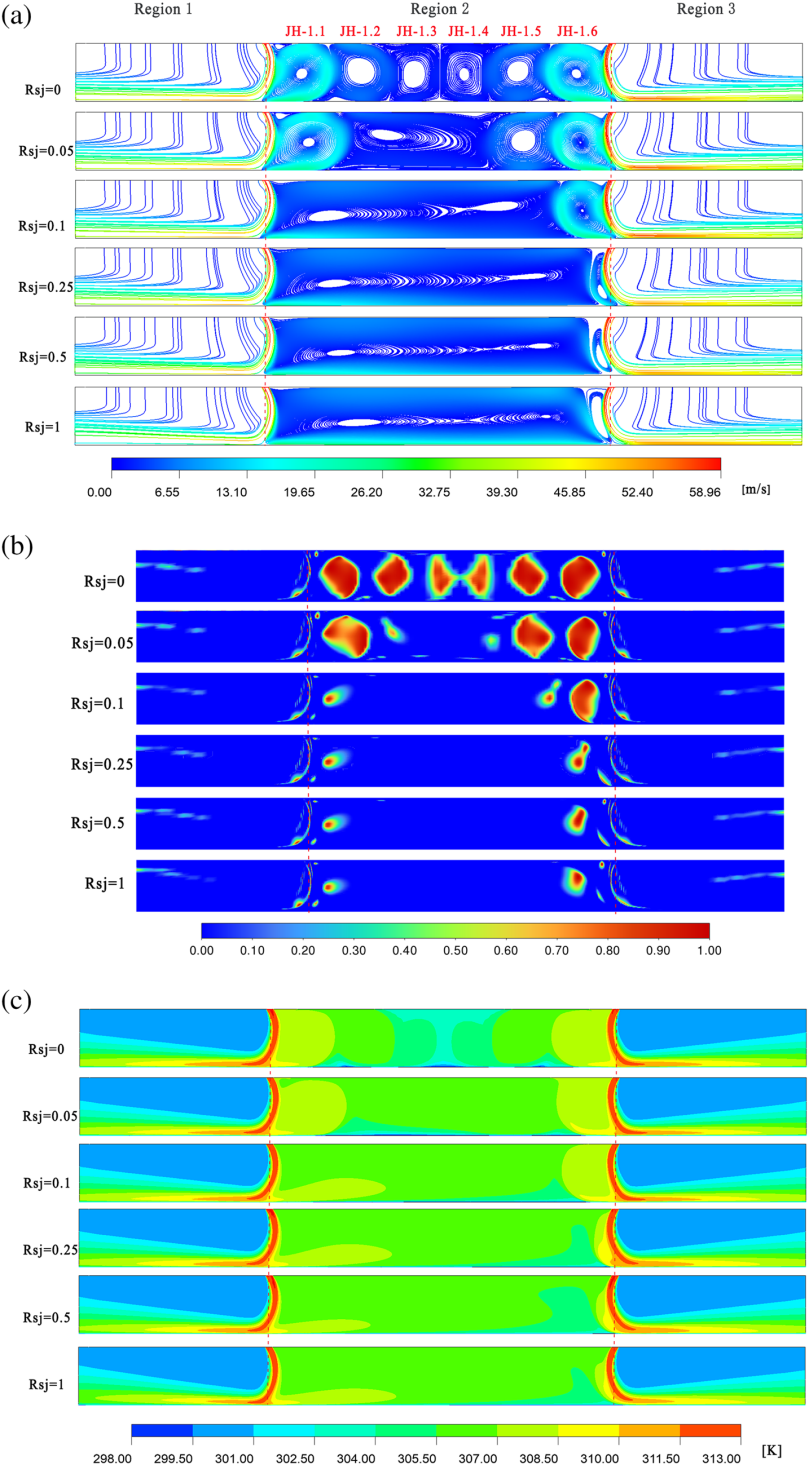
When $\alpha$ = 30°, $h/w$ = 8 , velocity flow diagram, $Q$ cloud diagram, and temperature distribution cloud diagram. Ansys Fluent2022, available at https://www.ansys.com/zh-cn/products/fluids/ansys-fluent, simulates and post-processes all pictures.

The velocity streamline variation for $\alpha$ = 30°, $h/w$ = 4, and $h/w$ = 8 is shown in Figs. 6a and 7a. In both scenarios, the airflow enters the flow field through the left and right air intakes and makes arc-shaped contact with the wall. A tiny amount of air flow will remain between the two air intakes as recirculation, creating a residency region, and eventually spread to the symmetric plane after the air flow strikes the wall. The majority of the air flow will exit via the air outlets on the left and right sides. Although the airflow velocity is higher near the entrance, it reaches its maximum when it meets the wall. Perfectly symmetrical flow fields and velocity fields are created on both sides of the symmetric surface under two operating conditions when the wall is immovable. The air flow velocity near the right wall is higher than that near the left wall due to the wall's increased movement speed.

The $Q$ cloud picture variation with varying $Rsj$ under two working conditions ($\alpha$ = 30°, $h/w$ = 4, and $h/w$ = 8) is depicted in Figs. 6b and 7b. Ten recirculations, designated JH-1, JH-2, JH-3, … JH-10 from left to right, were created in the dwell area between the two intakes under the parameters $h/w$ = 4 and $Rsj$ = 0. Since it is unable to distinguish between the JH2 and JH3, or JH4 and JH5 recirculations' intensities on a traditional scale, the $Q$ cloud map on a logarithmic scale is used to assess the intensities of the two nearby recirculations.

8 $$Q = \frac{1}{2}\left( {\Omega_{ij} \Omega_{ij} - S_{ij} S_{ij} } \right)$$

9 $$\Omega_{ij} = \frac{1}{2}\left( {\frac{{\partial u_{i} }}{{\partial x_{j} }} - \frac{{\partial u_{j} }}{{\partial x_{i} }}} \right)$$

10 $$S_{ij} = \frac{1}{2}\left( {\frac{{\partial u_{i} }}{{\partial x_{j} }} + \frac{{\partial u_{j} }}{{\partial x_{i} }}} \right)$$

The fluid particle' rotating angular velocity tensor is represented by $\Omega_{ij}$, while the angular deformation rate tensor is represented by $S_{ij}$. The presence of vortices in the fluid particle is indicated when their $Q$ value is higher than zero. The $Q$ cloud image makes it evident that the 10 recirculations' intensities diminish from the sides toward the center. The two recirculations closest to the symmetric plane, JH10 = JH1 > JH9 = JH2 > … > JH5 = JH6, have the weakest intensities. Since JH-1, JH-2, JH-9, and JH-10 have relatively strong recirculation strengths near the air inlet when $Rsj$ = 0.05, they are able to withstand the wall shear force that is produced in this situation. Because they are weak and unable to withstand the shear stress, the gyres JH-3, JH-4, JH-5, JH-6, and JH-7 close to the plane of symmetry combine to produce a sizable recirculation. The flow field at $Rsj$ = 0.05 is slightly altered when compared to $Rsj$ = 0. The two recirculations JH-1 and JH-2 at the left air intake are drawn together to form a big recirculation when $Rsj$ = 0.1 because they are unable to withstand the shear strain. JH-10 is pressed thinner, and JH-8 is squeezed upward at the same time. The flow field has changed considerably as of right now. When the nozzle flow field changes dramatically, as reported in the literature^16^, this is consistent with $Rsj$ < 0.25. JH1–JH9 are progressively drawn into a big recirculation as $Rsj$ gradually rises to 1, and two smaller recirculations emerge inside the large recirculation. While the flow field in the retention area becomes more uniform as $Rsj$ increases from 0.1 to 1, JH-10 does not vanish but instead flattens out over time.

Six recirculations, designated JH-1.1, JH-1.2, JH-1.3…JH1.6 from left to right, developed in the retention area between the two air intakes when $h/w$ = 8 and $Rsj$ = 0. The strength of these six recirculations diminishes from both sides to the middle, as can be seen in the $Q$ cloud map on the logarithmic scale. The two recirculations closest to the symmetric plane have the weakest intensities, which are JH-1.6 = JH-1.1 > JH-1.5 = JH-1.2 > JH-1.4 = JH-1.3. The two recirculations JH-1.1 and JH-1.6 near the air inlet are powerful enough to withstand the shear action of the wall, similar to the $h/w$ = 4 situation; therefore, their shapes and sizes essentially remain unchanged as $Rsj$ grows to 0.05. The flow field does not change considerably at this time because JH1.2, JH1.3, and JH1.4 near the symmetry plane have weak recirculation strength, which causes them to be dragged and merged into a huge recirculation. JH1.1–JH1.5 are gradually drawn into a massive recirculation at $Rsj$ = 0.1, and the flow field undergoes a major alteration. While JH-1.6 does not vanish with an increase in $Rsj$, it is gradually flattened as $Rsj$ increases to 1. JH1.1–JH1.5 are progressively drawn into a broad recirculation, and the flow field in the retention area becomes more uniform.

The cloud diagram of temperature field changes with $Rsj$ under the assumptions of $\alpha$ = 30°, $h/w$ = 4, and $h/w$ = 8 is displayed in Figs. 6c and 7c. The way that the temperature field changes with $Rsj$ and the flow field changes with $Rsj$ is comparable. While the main stream's outflow affects the temperature field in the jet zone on either side of the suspension nozzle, the diffusion of recirculation mostly determines the temperature field change in the retention zone. The local temperature inside the retention region and the recirculation site coincide under the $h/w$ = 4 condition when the wall surface is stationary. Because of the jet inclination angle, recirculation does not form in the vicinity of the symmetric plane; as a result, the flow field temperature in this area is low and nearly identical to that of the cold wall surface. When $Rsj$ = 0.05, recirculation and wall movement work together to cause temperature transfer, resulting in a progressive increase in temperature at the JH-3, JH-4, JH-5, JH-6, and JH-7 gyres. The temperature inside the retention area increased along with the recirculation's overall temperature as $Rsj$ increased from 0.1 to 1, eventually becoming uniform. The temperature cloud map, however, shows that even at $Rsj$ = 1, there was still a left-to-right temperature differential inside the retention area. When the wall is stationary and $h/w$ = 8, it is evident that the holdup area's local temperature almost coincides with the recirculation's location. This suggests that the diffusion of the recirculation is responsible for the temperature changes inside the holdup area. The flow field's local temperature rises when $Rsj$ = 0.05 due to the combined effects of the JH-1.2, JH-1.3, and JH-1.4 recirculations. The temperature inside the retention area increased along with the recirculation's overall temperature as $Rsj$ increased from 0.1 to 1, eventually becoming uniform. The overall temperature inside the retention zone under the $h/w$ = 8 condition is greater than that under the $h/w$ = 4 condition, which will be favorable to wall heat transfer. When $Rsj$ = 1, compared with the $h/w$ = 4 condition, the temperature field inside the retention zone is more uniform, and the temperature gradient is not obvious.

**Figure 8:**
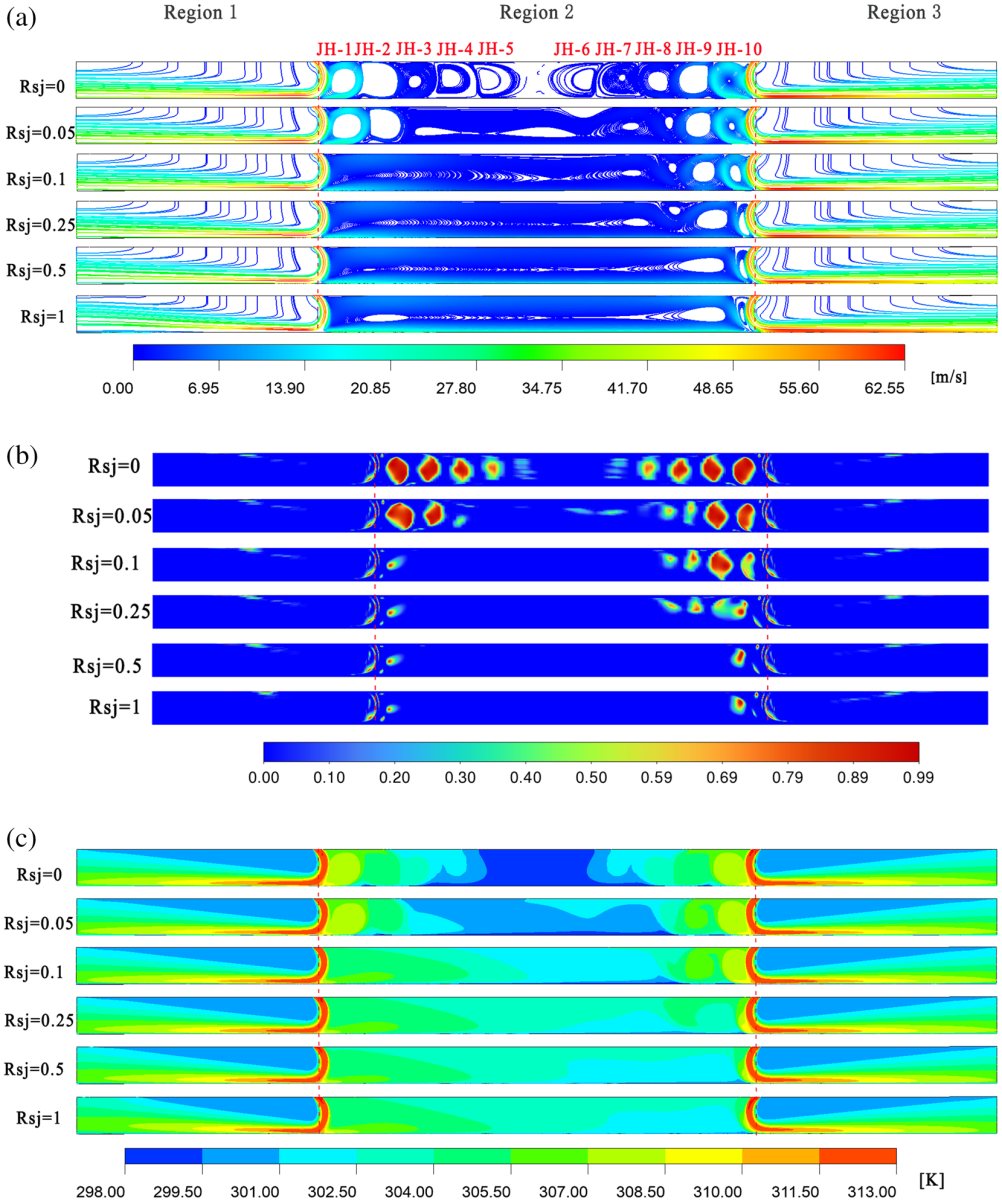
When $\alpha$ = 45°, $h/w$ = 4, velocity flow diagram, $Q$ cloud diagram, and temperature distribution cloud diagram. Ansys Fluent2022, available at https://www.ansys.com/zh-cn/products/fluids/ansys-fluent, simulates and post-processes all pictures.

**Figure 9:**
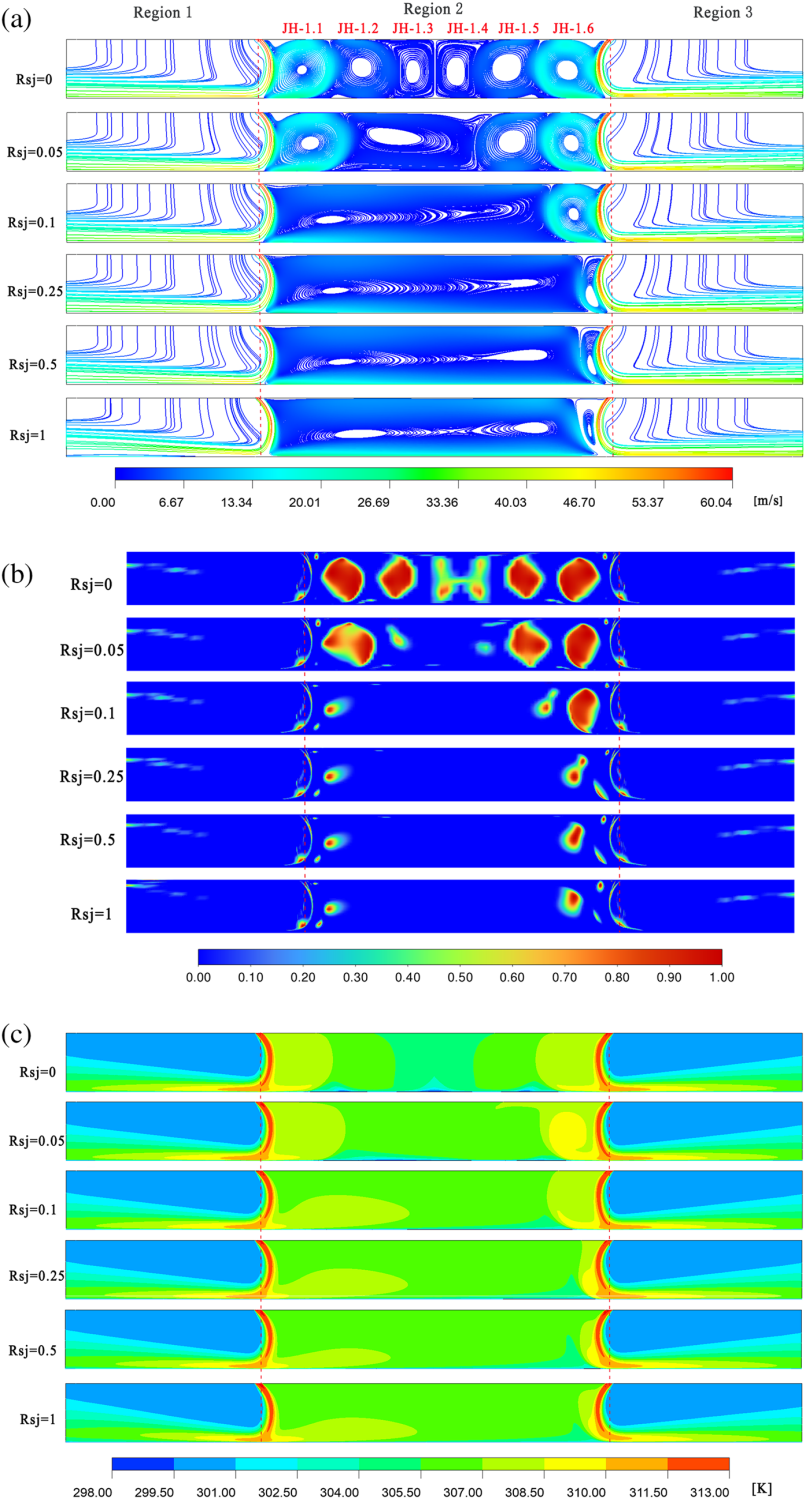
When $\alpha$ = 45°, $h/w$ = 8 , velocity flow diagram, $Q$ cloud diagram, and temperature distribution cloud diagram. Ansys Fluent2022, available at https://www.ansys.com/zh-cn/products/fluids/ansys-fluent, simulates and post-processes all pictures.

Figures 8a–c and 9a–c shows that, under the conditions of $\alpha$ = 45° and $h/w$ = 4, 10 recirculations still formed in the holdup area, and the area not covered by the recirculation near the symmetry plane was less than it was with $\alpha$ = 30° and $h/w$ = 4. Consequently, under the conditions of $\alpha$ = 45° and $h/w$ = 4, the low temperature region in the retention zone is less than that of the condition of $\alpha$ = 30° and $h/w$ = 4. This is true even when the wall is stationary. Nonetheless, under the two working conditions, the temperature fields of $\alpha$ = 45°, $h/w$ = 4, and $\alpha$ = 30°, $h/w$ = 4, are almost identical when $Rsj$ = 1, and a clear temperature gradient is still created.

The gyres JH1.3 and JH1.4 are squeezed at $\alpha$ = 45° and $h/w$ = 8, as opposed to $\alpha$ = 30° and $h/w$ = 8, and there is a significant energy exchange between them. As a result, under the conditions of $\alpha$ = 45° and $h/w$ = 8, the retention zone's temperature is typically higher while the wall is stationary than it is under the conditions of $\alpha$ = 30° and $h/w$ = 8. In the case of $Rsj$ = 1, as opposed to $h/w$ = 4, the temperature field within the retention zone under $h/w$ = 8 is more uniform, the temperature gradient is less noticeable, and the retention zone's overall temperature is higher under $h/w$ = 8 than it is under $h/w$ = 4.

**Figure 10:**
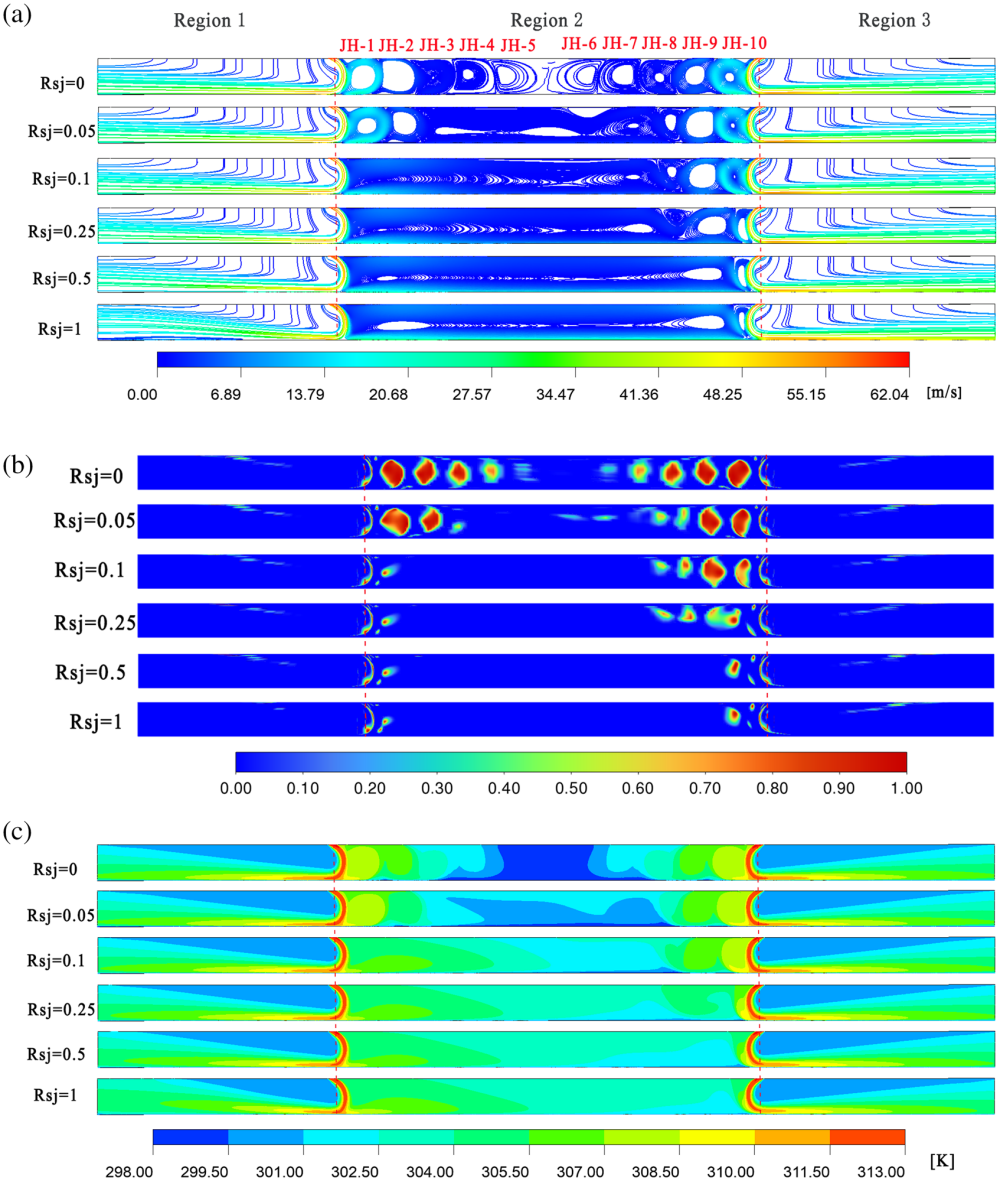
When $\alpha$ = 60°, $h/w$ = 4, velocity flow diagram, $Q$ cloud diagram, and temperature distribution cloud diagram. Ansys Fluent2022, available at https://www.ansys.com/zh-cn/products/fluids/ansys-fluent, simulates and post-processes all pictures.

**Figure 11:**
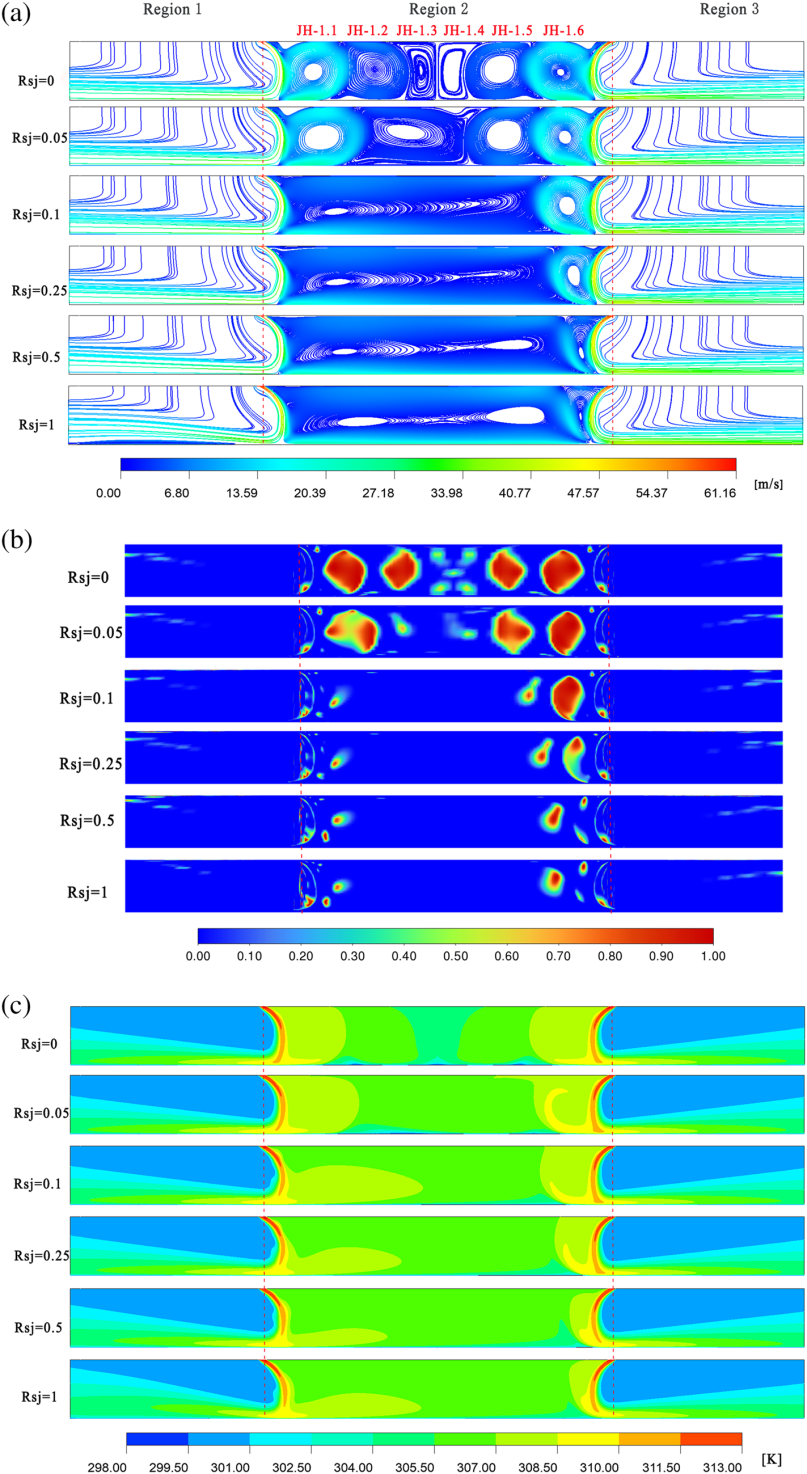
When $\alpha$ = 60°, $h/w$ = 8, velocity flow diagram, $Q$ cloud diagram, and temperature distribution cloud diagram. Ansys Fluent2022, available at https://www.ansys.com/zh-cn/products/fluids/ansys-fluent, simulates and post-processes all pictures.

As shown in Figs. 10a–c and 11a–c, under the conditions of $\alpha$ = 60° and $h/w$ = 4, 10 recirculations are still created in the holdup region, and the entire holdup area is covered by the recirculation, in contrast to $\alpha$ = 30° and 45°. Consequently, given the assumptions of $\alpha$ = 60° and $h/w$ = 4, the holdup area's low temperature area is lower when the wall is stationary.

Under $\alpha$ = 60° and $h/w$ = 8 circumstances, the recirculation of JH1.3 and JH1.4 is significantly constricted compared to $\alpha$ = 30° and 45°. As a result, under $\alpha$ = 60° and $h/w$ = 8 conditions, the retention zone's temperature is higher than it is under $\alpha$ = 45° and $h/w$ = 8 situations when the wall is stationary. The temperature field in the retention zone becomes more uniform as wall movement speed increases.

### Effects of $h/w$, $\alpha$ and $Rsj$ on heat transfer and pressure distribution

The Nusselt number ($Nu$), which denotes the wall's heat transfer capacity, can be used to express the wall's heat transfer intensity. The wall's local heat transmission intensity increases with increasing $Nu$ values.

11 $$Nu = \frac{{q_{conv} D}}{{k(T_{c} - T_{h} )}}$$

where $k$ is the thermal conductivity, $q_{conv}$ is the convective heat flux on the wall surface, $T_{c}$ and $T_{h}$ are the wall surface temperature and reference temperature, respectively, and the reference temperature can generally be selected as the fluid temperature ($T$) at the jet inlet or the adiabatic wall temperature.

**Figure 12:**
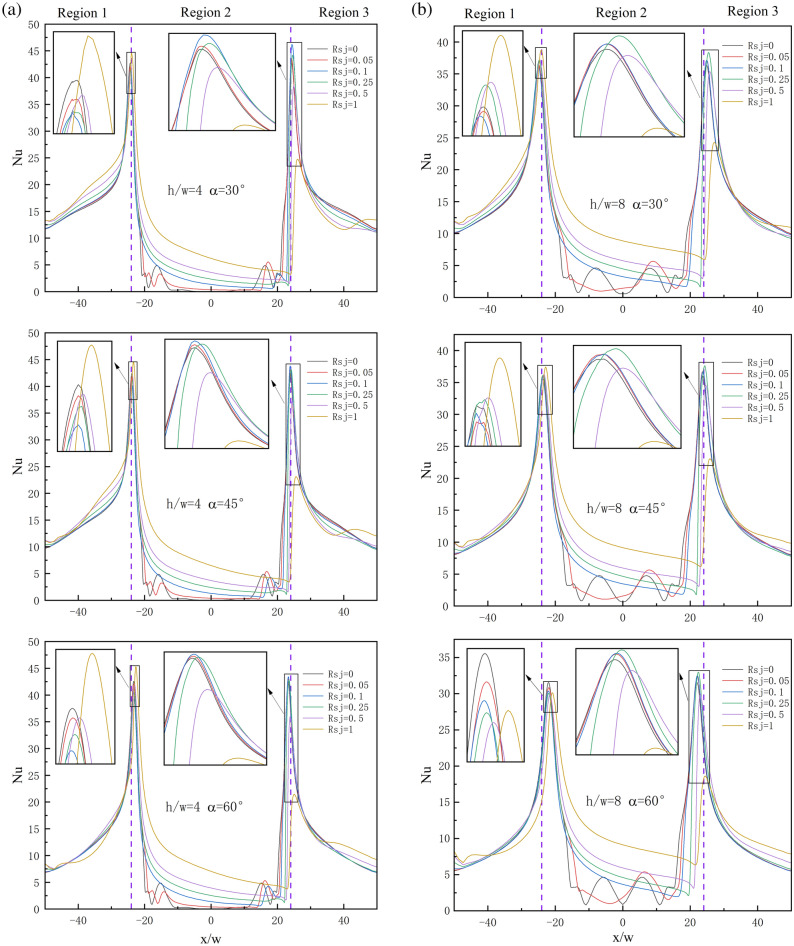
Local $Nu$ distribution on the wall under different working conditions.

The local $Nu$ distribution curve of the suspension nozzle, as depicted in Fig. 12a,b, can be loosely categorized into three regions: -50 < $x/w$ < -24, -24 < $x/w$ < 24, and 24 < $x/w$ < 50. These regions are referred to as region 1, region 2, and region 3, respectively, since they are arranged left to right. Compared to regions 1 and 3, region 2 is significantly more impacted by $Rsj$. The figure displays many peaks for each $Nu$ curve, with the largest peak value being close to ± 24. When it is 0 < $Rsj$ < 0.1 in the observable range, the left peak value falls with an increase in $Rsj$; when it is 0.25 < $Rsj$ < 1, it grows with an increase in $Rsj$. In the end, the value of the left peak under $Rsj$ = 1 is higher than that under $Rsj$ = 0. When 0 < $Rsj$ < 0.1, the right peak value increases with an increase in $Rsj$; when 0.25 < $Rsj$ < 1, it drops with an increase in $Rsj$. Lastly, under $Rsj$ = 1, the right peak value is smaller than under $Rsj$ = 0.

When the wall is stationary under the condition $h/w$ = 4, for any $\alpha$, the recirculation intensity at the symmetric plane is very weak, the temperature here is nearly equal to the wall temperature, and there is almost no wall heat transfer. These loops are placed around the left and right intakes, where there is considerable recirculation intensity. As a result, the $Nu$ curve shows four local peaks in this area. With the combination of JH-4, JH-5, JH-6, and JH-7 recirculations in region 2, the wall heat transfer near the symmetric surface increases when $Rsj$ = 0.05, and the local peak value in area 2 on the $Nu$ curve becomes smooth. The local peak on the left side of the $Nu$ curve in region 2 vanishes, but the local peak on the right side stays when $Rsj$ = 0.1 because the recirculation close to the left inlet is also combined into a larger recirculation. When 0.1 < $Rsj$ < 1, area 2's wall heat transmission progressively rises as recirculation continues to solidify. The peak value at the recirculation JH-10's point on the $Nu$ curve has always existed since the recirculation JH-10 has always existed.

Since zone 2 is fully covered by recirculation under $h/w$ = 8 for any $\alpha$, when the wall is stationary, the wall heat transfer under this condition is higher than that under $h/w$ = 4. When JH-1.2, JH-1.3, and JH-1.4 are combined, the local peak in region 2 becomes smooth at $Rsj$ = 0.05. The local peak on the left side of the $Nu$ curve in region 2 vanishes, but the local peak on the right side stays when $Rsj$ = 0.1 because the recirculation close to the left inlet is also combined into a larger recirculation. When 0.1 < $Rsj$ < 1, area 2's wall heat transmission progressively rises as recirculation continues to solidify. Overall, the heat transfer intensity in zone 2 is greatly increased, but the peak value of the left and right sides of the $h/w$ = 8 condition is reduced when compared to the $h/w$ = 4 condition.

$\alpha$ hardly affects the wall surface's local $Nu$ within the observed range. It is noteworthy, therefore, that the peak values on the left and right sides progressively approach the symmetric plane as the jet inclination increases. This is consistent with the change law of the jet stationary point with the angle in the flow field.

The wall pressure, which represents the wall's suspension capacity, can be expressed using the pressure coefficient ($Cp$). The local pressure of the wall increases with an increasing $Cp$ value.

12 $$Cp = \frac{2P}{{\rho u_{j}^{2} }}$$

where $P$ is the local pressure of the wall, $u_{j}$ is the jet inlet velocity.

**Figure 13:**
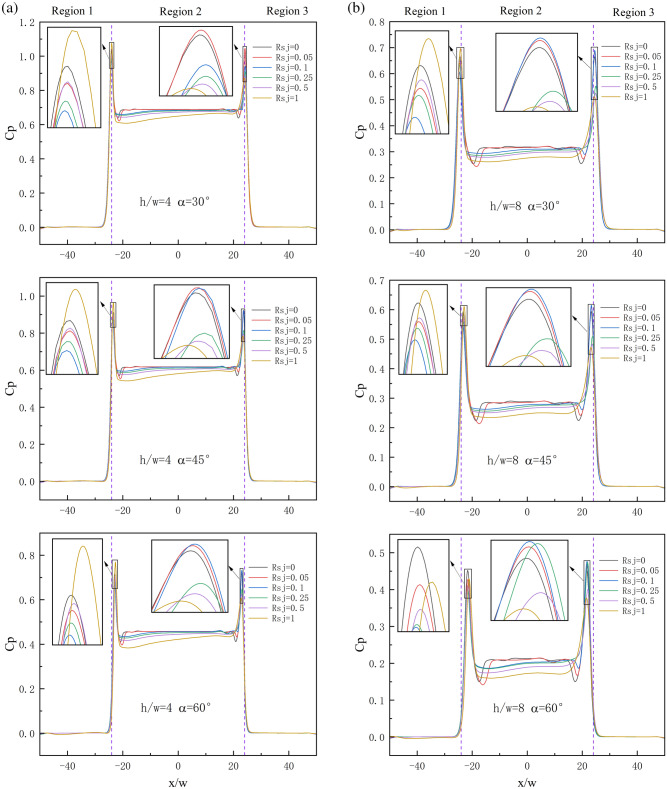
Local $Cp$ distribution on the wall under different working conditions.

The formation of an "air cushion" between the two air inlets of the suspension nozzle facilitates the suspension of the wall, as seen by the local wall $Cp$ figure in Fig. 13a,b. Any $h/w$, any $\alpha$, or any $Rsj$ circumstances have essentially no effect on the $Cp$ values of region 1 and region 3 within the visible range.

The highest peak value is always seen at the left and right stagnation points. When 0 < $Rsj$ < 0.1, the left peak value drops with an increase in $Rsj$; when 0.25 < $Rsj$ < 1, it increases with an increase in $Rsj$. Lastly, when $Rsj$ = 1, the left peak value is higher than the right peak value. When 0 < $Rsj$ < 0.1, the right peak value increases with an increase in $Rsj$; when 0.25 < $Rsj$ < 1, it drops with an increase in $Rsj$. Last but not least, the right peak value is lower when $Rsj$ = 1 than it is when $Rsj$ = 0. In area 2, $Cp$ gradually drops as $Rsj$ rises because the pressure of the "air cushion" is weakened by the merging of recirculation.

The $Cp$ value of area 2 is much bigger than that under the $h/w$ = 8 condition, and the maximum value of any $Cp$ curve under the $h/w$ = 4 condition is greater than that under the $h/w$ = 8 condition. This suggests that the more advantageous the wall's suspending effect, the smaller the $h/w$.

$Cp$ progressively drops as a rises under $h/w$ = 4 and $h/w$ = 8, suggesting that an increase in $\alpha$ is not favorable for the wall's suspension.

In order to more accurately depict the heat transfer effect and suspension capacity of the entire wall surface, $\overline{Nu}$ and $\overline{Cp}$ are employed to represent the average heat transfer intensity and average pressure coefficient of the entire wall surface.

13 $$\overline{Nu} = \frac{1}{L}\int\limits_{ - L/2}^{L/2} {Nu(x)} dx$$

14 $$\overline{Cp} = \frac{1}{L}\int\limits_{ - L/2}^{L/2} {C_{p} (x)} dx$$

**Figure 14:**
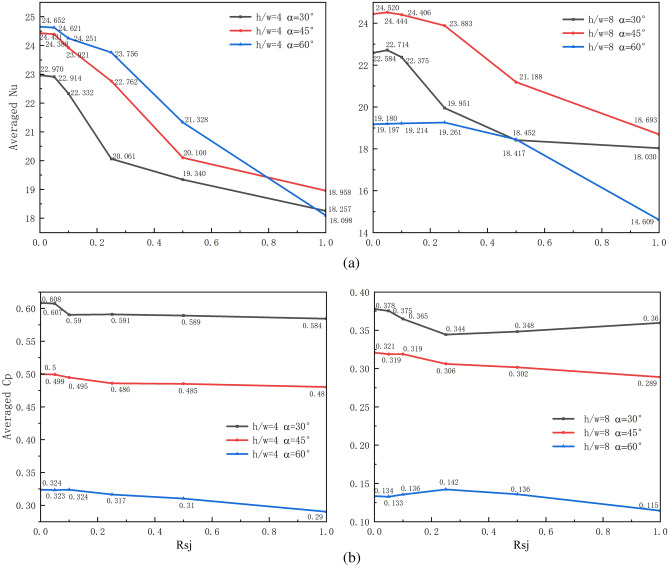
$\overline{Nu}$ and $\overline{Cp}$ distribution on the wall under different working conditions.

The $\overline{Nu}$ and $\overline{Cp}$ distribution on the wall under varied operating conditions is depicted in Fig. 14a,b. $\overline{Nu}$ reduces greatly with an increase in $Rsj$ when $h/w$ and $\alpha$ remain constant, but $\overline{Cp}$ decreases somewhat. The largest reduction in $\overline{Nu}$ is 26.58% under $h/w$ = 4 and $\alpha$ = 60°. At $\alpha$ = 30° and $h/w$ = 8, the $\overline{Nu}$ reduction is at least 17.95%. The $\overline{Cp}$ reduction is 14.23% at $h/w$ = 8 and $\alpha$ = 60°. The $\overline{Cp}$ reduction is the smallest at 3.95% under $h/w$ = 4, $\alpha$ = 30°, and 45°. Compared to $\overline{Cp}$, $\overline{Nu}$ has decreased by a significantly larger amount.

## Conclusions

This paper examines the steady-state characteristics of heat transfer and the pressure of the suspension nozzle while treating the flow field as a two-dimensional turbulent flow. The rectangular flow field is composed of an adiabatic wall that runs parallel to the moving wall and two slit entrances at either end of the adiabatic wall. The turbulence computation makes use of the SST $k - \omega$ turbulence model. The issue parameters include a Reynolds number (${\text{Re}}$) of 6000, a wall-to-jet velocity ratio ($Rsj$) of 0–1, a jet inclination ($\alpha$) of 30°, 45°, and 60°, and separation spacing to slit width ratios ($h/w$) of 4 and 8. Both qualitative and quantitative analyses are conducted on the distribution of the flow field, temperature field, local Nusselt number, local pressure coefficient, average Nusselt number, and average pressure coefficient under various combination conditions.

1. $\alpha$ has a minimal effect on the flow field and only affects the recirculation distribution within the retention area, which in turn affects the temperature distribution across the retention zone. Assuming that $Rsj$ and $h/w$ remain constant, the temperature inside the retention region is typically greater with a larger $\alpha$. $\alpha$ hardly has an effect on the wall's heat-transfer capacity, but it does change where the jet strikes the wall, which influences the location of the wall's stagnation point. As $\alpha$ rises, $Cp$ gradually decreases, which is bad for the suspension of the wall.
2. $h/w$ has a major effect on the flow field, and the quantity of $h/w$ determines the number of recirculations inside the retention zone. Although there is no direct correlation between the quantity of recirculation and the change in temperature, heat exchange will still take place in the retention zone as long as there is recirculation coverage. When the $h/w$ is larger, the recirculation in the retention zone is more completely developed, which enhances the wall's capacity to transmit heat. However, $Cp$ sharply falls as $h/w$ rises, which is bad for the wall's suspension.
3. The flow field is most influenced by $Rsj$, and when the flow field varies greatly, $Rsj$ is 0.1. The diffusion of recirculation plays a major role in determining the temperature change inside the retention area when $Rsj$ is modest. When $Rsj$ is high, wall movement's driving effect on air flow determines how much the retention area's temperature changes. The maximum peak change on the $Nu$ curve and the maximum peak change on the $Cp$ curve both have an inflection point of 0.1. With the recirculation in the retention region merging, the larger the $Rsj$, the greater the $Nu$ of region 2, and the stronger the wall's ability to transfer heat. But the recirculation's merging also lessens the "air cushion's" pressure, which lowers the wall's ability to support suspension.
4. It is discovered that the suspension nozzle's $\overline{Nu}$ and $\overline{Cp}$ change with $\alpha$, $h/w$, and $Rsj$. When $\alpha$ and $h/w$ is constant, $\overline{Cp}$ reduces slightly while $\overline{Nu}$ decreases dramatically as $Rsj$ increases. The largest reduction of $\overline{Nu}$ in the measured $Rsj$ range is 26.58% under $h/w$ = 4 and $\alpha$ = 60°, and the maximum reduction of $\overline{Cp}$ is 14.23% under $h/w$ = 8 and $\alpha$ = 60°.

## Data Availability

The datasets supporting this article have been uploaded as part of the Supplementary Material.
